# Optimization of Sensors Data Transmission Paths for Pest Monitoring Based on Intelligent Algorithms

**DOI:** 10.3390/bios12110948

**Published:** 2022-11-01

**Authors:** Yuyang Lian, Aqiang Wang, Sihua Peng, Jingjing Jia, Liang Zong, Xiaofeng Yang, Jinlei Li, Rongjiao Zheng, Shuyan Yang, Jianjun Liao, Shihao Zhou

**Affiliations:** 1 Sanya Nanfan Research Institute of Hainan University, Sanya 572025, China; 2 Key Laboratory of Germplasm Resources Biology of Tropical Special Ornamental Plants of Hainan Province, College of Forestry, Hainan University, Haikou 570228, China; 3 Hainan Key Laboratory for Control of Plant Diseases and Insect Pests, Haikou 571199, China; 4 College of Information Engineering, Shaoyang University, Shaoyang 422000, China

**Keywords:** pest monitoring, sensors, data transmission, genetic algorithms, particle swarm optimization, simulated annealing

## Abstract

The harm of agricultural pests presents a remarkable effect on the quality and safety of edible farm products and the monitoring and identification of agricultural pests based on the Internet of Things (IoT) produce a large amount of data to be transmitted. To achieve efficient and real-time transmission of the sensors’ data for pest monitoring, this paper selects 235 geographic coordinates of agricultural pest monitoring points and uses genetic algorithm (GA), particle swarm optimization (PSO), and simulated annealing (SA) to optimize the data transmission paths of sensors. The three intelligent algorithms are simulated by MATLAB software. The results show that the optimized path based on PSO can make the shortest time used for transmitting data, and its corresponding minimum time is 4.868012 s. This study can provide a reference for improving the transmission efficiency of agricultural pest monitoring data, provide a guarantee for developing real-time and effective pest control strategies, and further reduce the threat of pest damage to the safety of farm products.

## 1. Introduction

The use of large amounts of fossil fuels has accelerated global warming since the 20th century, and atmospheric temperatures and sea levels are rising at an unprecedented rate [1]. It is expected that the global average temperature will rise by 1.5–4.5 °C by the end of this century [2]. Insects are typically poikilotherm, and temperature is the most important environmental factor affecting insect population dynamics. Climate warming present a series of influences on pests, such as expanding the range of fitness zones [3], increasing the survival rate of overwintering, increasing the number of generations [4], and increasing the risk of spreading crop diseases. These effects may cause sudden local outbreaks or migrations of pest populations, reduce crop yields, affect normal crop growth and development, and even cause massive crop mortality and severe crop failure, thus presenting a serious threat to the quality and safety of agricultural products. Once crop yields are severely affected, it can cause local crop supply and demand to be relatively tight, making the price of food products remarkable high and hindering the steady improvement of a region’s agricultural economy. This means that the agricultural pests cause huge economic losses as well as presenting a remarkable threat to food safety [5,6]. Only through effective monitoring methods to achieve early warning of pest occurrence can we reduce agricultural losses and ensure the safety of edible farm products.

Traditionally, the acquisition of pest information is mainly performed by manual field survey and identification statistics, and the monitoring of pest situations is based on the manual survey, which is a method with high workload, low efficiency, poor reliability, and low accuracy [7,8]. Agricultural pest monitoring and identification using the Internet of Things (IoT) has become an important component of the agricultural pest control category with the advent of Industry 4.0. Combining agriculture with modern electronic information industries such as communications and sensors has greatly improved the efficiency and accuracy of pest monitoring and identification. Gassoumi et al. designed a computer vision-based insect classification and identification system for cotton fields, using an artificial neural network approach to classify 12 insect species in cotton fields, the recognition rate of 11 insect species reached more than 90%, and the recognition rate of only one insect was 72% [9]. Based on the existing light trapping technology, an automatic pest collection device that can be used for cruciferous major insect pest detection was studied and designed. Using this device, images of cruciferous major pests can be collected, flipped, and vibrated according to the real-time situation of pest images, which effectively improves the quality of image collection and recognition accuracy, and the recognition error is less than 5% [10]. Based on a wireless network image sensors system, a pest remote automatic monitoring and early warning system was designed, which uses the technique of background differencing to achieve pest counting and sends an early warning message when the pest density is greater than a threshold value [11]. Pest monitoring and identification technology are continuously optimized and the accuracy is continuously improved, but multiple monitoring points are often set up in the process of agricultural pest monitoring in the field. The network will be blocked when the number of monitoring points increases or during the peak of pest occurrence, which will delay or collapse the data transmission, thus limiting the real-time monitoring of agricultural pests. As a result, the information on the species and quantity of pests cannot be obtained in time, and effective pest control strategies cannot be formulated. Therefore, it is urgent to increase research on the optimization of sensors’ data transmission paths for pest monitoring to achieve efficient and real-time transmission of multi-point data in the process of pest monitoring.

**Figure 1:**
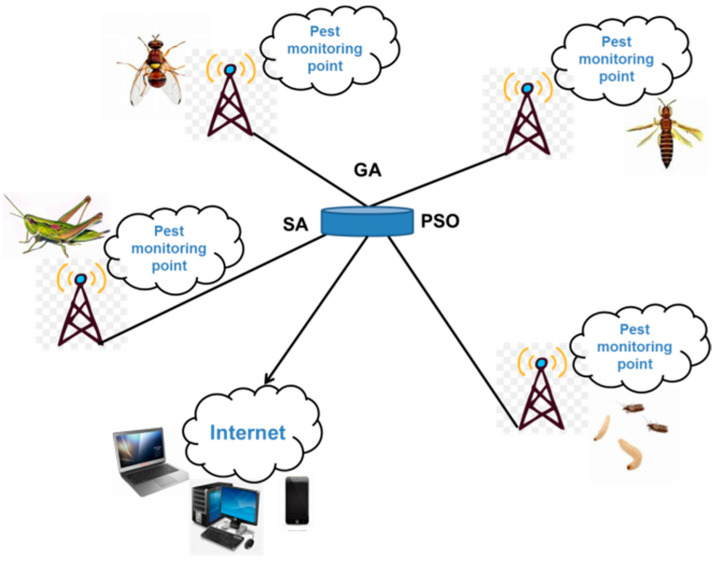
The sensors’ data transmission paths for multiple pest monitoring points are optimized based on genetic algorithm, particle swarm optimization, and simulated annealing.

Genetic algorithm (GA), swarm optimization (PSO), and simulated annealing (SA) are all bionic intelligence algorithms which are widely used in industry, agriculture, and medicine to find optimal solutions [11]. GA, also known as the evolutionary algorithm (EA), is a heuristic algorithm based on the process of biological evolution. Its main feature is the selection of the solution to the problem based on the “survival of the fittest” or “competition” method of biological evolution [12]. GA is based on all individuals in a population and uses randomization techniques to guide an efficient search of coded parameter space. Among them, selection, crossover, and mutation constitute the genetic operations of GA; five elements, namely, parameter encoding, initial population setting, design of fitness function, design of genetic operations, and setting of control parameters, form the core of GA [13]. GA has been widely used in the fields of combinatorial optimization, machine learning, signal processing, adaptive control, and artificial life, and it is one of the key techniques in modern relevant intelligent computing [14]. Zhang tested the performance of the improved GA on the transmission path of wireless Mesh network through simulation to optimize the transmission path of marine data and improve the efficiency of marine data transmission [15]. PSO, also known as the birds’ flock foraging algorithm, is a new EA developed by Kennedy and Eberhart et al. in recent years. The PSO starts from a random solution, finds the optimal solution by iteration, evaluates the quality of the solution by fitness, and follows the current searched optimal value to find the global optimal. This algorithm has attracted the attention of academics for its easy implementation, high accuracy, and fast convergence, and has demonstrated its superiority in solving practical problems [16]. Ma et al. applied the PSO to the medical field, and the research based on the PSO will use a multi-PSO for path planning to effectively reduce the path search range and improve the path search speed [17]. SA is a general probabilistic heuristic algorithm for combinatorial optimization problems to find a global optimal solution and an approximate optimal solution in a large global search space [18]. The SA is often used for searching in discrete spaces; it will be more effective than the exhaustive method for some problems because it aims to find a good solution in an acceptable time, rather than the best solution [19]. Based on the SA, Liu et al. proposed an optimal design method for the heat extraction section of the energy tunnel, which effectively improved the heat transfer efficiency and reduced the construction cost [20]. The sensors’ data transmission paths for pest monitoring are optimized based on GA, PSO, and SA in our study (Figure 1), and the data transmission speed is compared and analyzed after the optimized paths, which improves the transmission efficiency of agricultural pest monitoring data, provides a guarantee for the formulation of effective agricultural pest control strategies, and reduces the threat of pest damage to the quality and safety of farm products.

## 2. Methods

In this paper, 235 geographic location coordinates of agricultural pest monitoring points are selected, and the sensors’ data transmission paths for pest monitoring points are optimized and analyzed based on GA, PSO, and SA, and the optimal transmission path and transmission time are determined.

### 2.1. Genetic Algorithm

The GA is used to analyze the prediction of their weights.

Step1. Encode the data:

(1) $$G_{k}=\left[V_{1},V_{2},\ldots ,V_{t},\ldots ,V_{T}\right]={\left[R_{1},R_{2},\ldots ,R_{i},\ldots ,R_{n}\right]}^{T}=\left[\begin{array}{cccccc} p_{11} & p_{12} & \cdots & p_{1t} & \cdots & p_{1T}\\ p_{21} & p_{22} & \cdots & p_{2t} & \cdots & p_{2T}\\ \vdots & \vdots & \cdots & \vdots & \cdots & \vdots \\ p_{i1} & p_{i2} & \cdots & p_{it} & \cdots & p_{iT}\\ \vdots & \vdots & \cdots & \vdots & \cdots & \vdots \\ p_{n1} & p_{n2} & \cdots & p_{nt} & \cdots & p_{nT} \end{array}\right]$$

(2) $$u_{it}=\{\begin{array}{cc} 0, & p_{it}=0\\ 1, & \mathrm{other} \end{array}$$

Step2. Coding during initialization of genetic populations:

(3) $$R=\left[r_{1},r_{2},\ldots ,r_{i},\ldots ,r_{n}\right]\;i=1,2,\ldots ,n$$

(4) $$per=\left[per_{1},per_{2},\ldots ,per_{i},\ldots ,per_{n}\right]$$

(5) $$\begin{array}{ccc} per_{i} & = & \frac{r_{i}}{\sum_{i=1}^{n}r_{i}}\;\;\;i=1,2,\ldots ,n\\ V_{t} & = & {\left[p_{1t},p_{2t},\ldots ,p_{it},\ldots ,p_{nt}\right]}^{T} \end{array}$$

Continue to optimize and simulate it.

Step3. Adjustment for each individual adaptation:

(6) $$p_{it}{}^{1}=\{\begin{array}{cc} p_{i\max}, & p_{it}>p_{i\max}\\ p_{it}, & p_{i\min}\leq p_{it}\leq p_{i\max}\\ p_{i\min}, & \lambda p_{i\min}\leq p_{it}\leq p_{i\max}\\ 0, & \mathrm{other} \end{array}$$

Step4. The function takes the following form:

(7) $$f_{itness}(G_{k})=\frac{A}{\left(F+\sum_{i}^{n}m\delta S_{i}\right)}$$

Step5. The selection probability is calculated by:

(8) $$P(x_{i})=\frac{f_{itness}(x_{i})}{\sum_{j=1}^{n}f_{itness}(x_{j})}$$

(9) $$q_{i}=\sum_{j=1}^{i}P(x_{j})$$

Step6. Cross mutation and offspring combination were carried out:

(10) $$\begin{array}{l} C_{1}\;=\;G^{C1}\;=\;\left[V_{1}{}^{C1},V_{2}{}^{C1},\ldots ,V_{t}{}^{C1},\ldots ,V_{T}{}^{C1}\right]\\ C2\;=\;G^{C2}\;=\;\left[V_{1}{}^{C2},V_{2}{}^{C2},\ldots ,V_{t}{}^{C2},\ldots ,V_{T}{}^{C2}\right] \end{array}$$

The specific operation process is:

(11) $$\begin{array}{l} D_{1}\;=\;\left[V_{1}{}^{C1},V_{2}{}^{C1},\ldots ,V_{j-1}{}^{C1},(1-\alpha )V_{j}{}^{C1}+\alpha V_{j}{}^{C2},V_{j+1}{}^{C1},\ldots ,V_{T}{}^{C1}\right]\\ D_{2}\;=\;\left[V_{1}{}^{C2},V_{2}{}^{C2},\ldots ,V_{j-1}{}^{C2},\alpha V_{j}{}^{C1}+(1-\alpha )V_{j}{}^{C2},V_{j+1}{}^{C2},\ldots ,V_{T}{}^{C2}\right] \end{array}$$

(12) $$\begin{array}{l} O_{1}=\max=\{f_{itness}(C_{1}),f_{itness}(D_{1})\}\\ O_{2}=\max=\{f_{itness}(C_{2}),f_{itness}(D_{2})\} \end{array}$$

Step7. Individual crossover through Step 6:

(13) $$\begin{array}{l} E_{1}\;=\;\left[V_{1}{}^{O1},V_{2}{}^{O1},\ldots ,V_{t}{}^{O1},\ldots ,V_{T}{}^{O1}\right]\\ E_{2}\;=\;\left[V_{1}{}^{O2},V_{2}{}^{O2},\ldots ,V_{t}{}^{O2},\ldots ,V_{T}{}^{O2}\right] \end{array}$$

$$\eta ,(\eta =\{\begin{array}{cc} 1, & \mathrm{Individuals}\;\mathrm{mutated}\;\\ 0, & \;\mathrm{Individuals}\;\mathrm{did}\;\mathrm{not}\;\mathrm{mutate} \end{array})$$

(14) $$\begin{array}{l} H_{1}\;=\;\left[V_{1}{}^{O1},V_{2}{}^{O1},\ldots ,\eta \beta V_{\gamma }{}^{O1}+(1-\eta )V_{\gamma }{}^{O1},V_{\gamma +1}{}^{O1},\ldots ,V_{T}{}^{O1}\right]\\ H_{2}\;=\;\left[V_{1}{}^{O2},V_{2}{}^{O2},\ldots ,\eta \beta V_{\gamma }{}^{O2}+(1-\eta )V_{\gamma }{}^{O2},V_{\gamma +1}{}^{O2},\ldots ,V_{T}{}^{O2}\right] \end{array}$$

(15) $$\begin{array}{l} I_{1}\;=\;\max\;=\;\{f_{itness}(E_{1}),f_{itness}(H_{1})\}\\ I_{2}\;=\;\max\;=\;\{f_{itness}(E_{2}),f_{itness}(H_{2})\} \end{array}$$

### 2.2. Particle Swarm Optimization

In the PSO algorithm, the particle population is searched in an n-dimensional space, where the position of each particle $X_{i}$ represents a solution to the problem, and the particle searches for a solution by continuously updating its position.

The position of the *i*-th particle at moment *t* is denoted by $X_{i.t}=[x_{i.t.1},x_{i.t.2},\cdots ,x_{i.t.n}]$.

The velocity of the first particle at moment *t* is denoted by $V_{i.t}=[v_{i.t.1},v_{i.t.2},\cdots ,v_{i.t.n}]$.

The first particle remembers at each moment the best position it searched for before moment *t*, denoted as $P_{t}$, and the best position searched for by the whole particle population, denoted as $P_{g}$.

**Figure 2:**
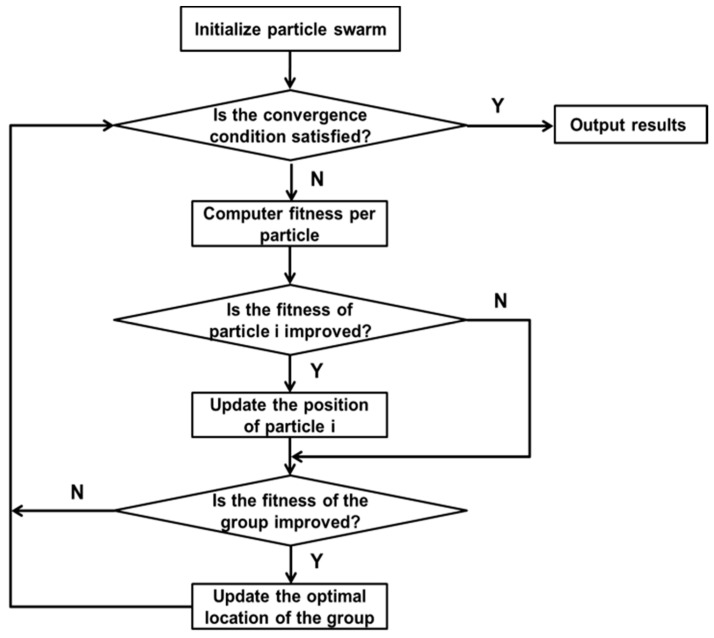
Description of particle swarm optimization.

The velocity $V_{it}$ of each particle at moment *t* is influenced by its own optimal position and the population optimal position, and the velocity and position of each particle are updated according to the following equation:

$$\begin{array}{l} V_{i,t}=\omega V_{i,t-1}+c_{1}r_{1}(P_{i}-X_{i})+c_{2}r_{2}(P_{g}-X_{i})\\ V_{i,t}=V_{i,t-1}+V_{i,t} \end{array}$$

where ω is called the power inertia factor, $c_{1},c_{2}$ is a positive acceleration constant, and $r_{1},r_{2}$ is a random number uniformly distributed between 0 and 1. The corresponding algorithm flowchart of the PSO is shown in Figure 2

### 2.3. Simulated Annealing

**Figure 3:**
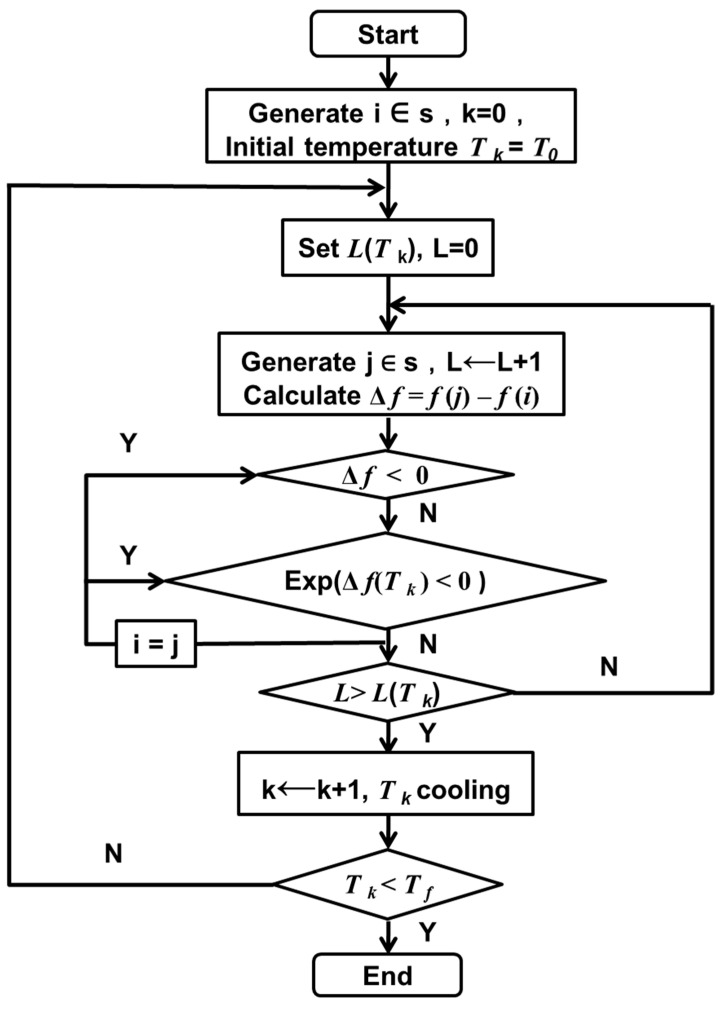
Description of simulated annealing.

The optimization of sensors’ data transmission paths for pest monitoring based on SA first determines the initial temperature, randomly selects an initial state and examines the objective function value of that state, attaches a small perturbation to the current state and calculates the objective function value of the new state, and accepts the better point with probability 1 and the worse point with some probability Pr as the current point until the system cools down. The SA is able to converge to the global optimum with probability 1 under the condition that the initial temperature is high enough and the temperature decreases slowly enough. The SA process of IoT monitoring data transmission is completed, and the regulation of monitoring network path optimization is finally realized. The corresponding algorithm flow chart is shown in Figure 3.

## 3. Result

### 3.1. Genetic Algorithm

**Figure 4:**
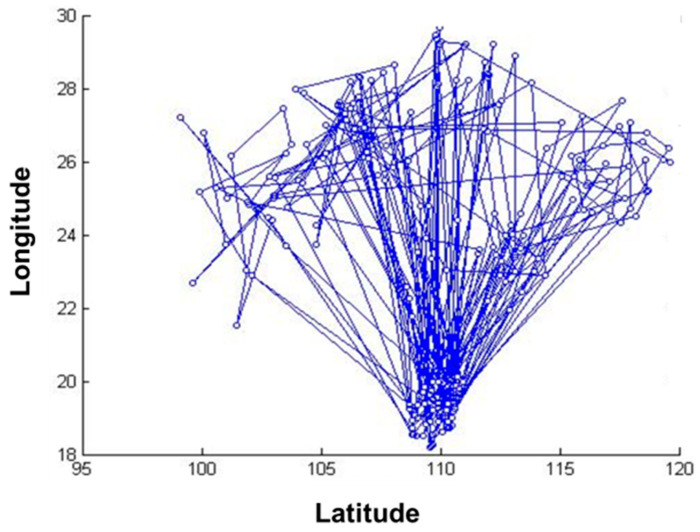
Running results of the genetic algorithm.

**Figure 5:**
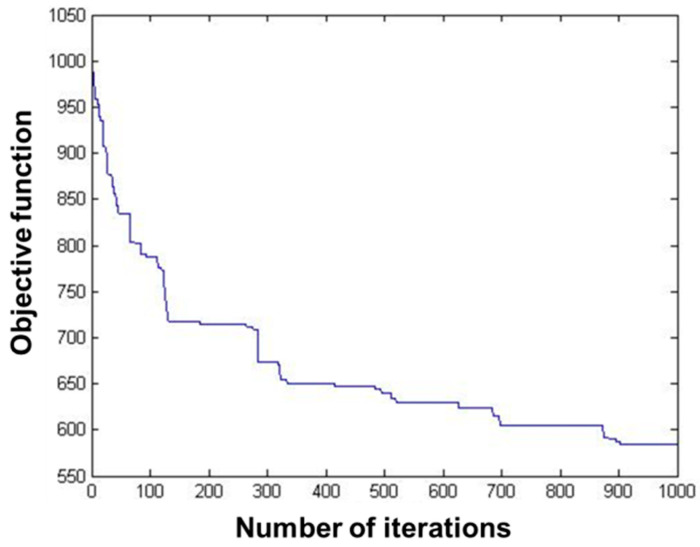
Convergence trajectory of the genetic algorithm.

The data of 235 pest monitoring points were imported into MATLAB software (MathWorks, MA, USA), and the GA was used for optimization analysis, and the results are shown in Figure 4 and Figure 5. In order to test whether the GA-based pest monitoring data reached the global minimum, the total number of iterations was set to 1000. The value of the objective function gradually decreased, and the number of transients of its convergence trajectory increases with the increase in the number of iterations, resulting in many local minima, which reach the global minimum at about 900 iterations.

### 3.2. Particle Swarm Optimization

**Figure 6:**
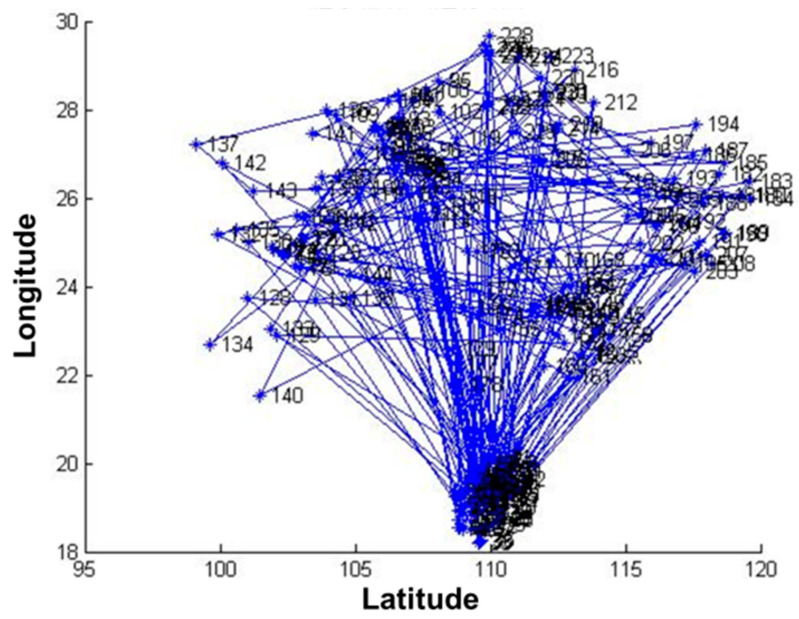
Running results of particle swarm optimization.

**Figure 7:**
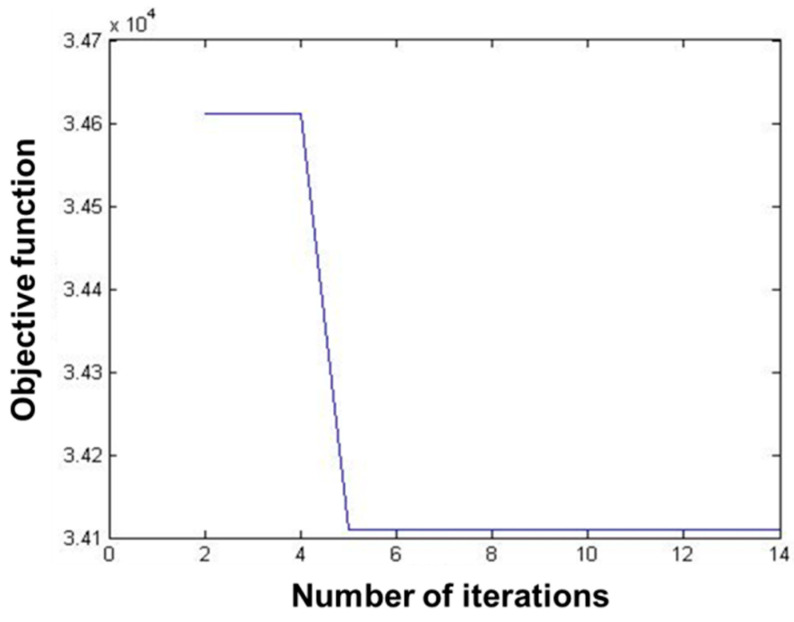
Convergence trajectory of particle swarm optimization.

The data of 235 pest monitoring points were imported into MATLAB software, and the PSO was used for optimization analysis, and the results are shown in Figure 6 and Figure 7. The total number of iterations was set to 14, and the value of the objective function decreased with the increase in the number of iterations, and its convergence trajectory started to converge rapidly at the number of iterations of four, and reached the global minimum at about five iterations.

### 3.3. Simulated Annealing

**Figure 8:**
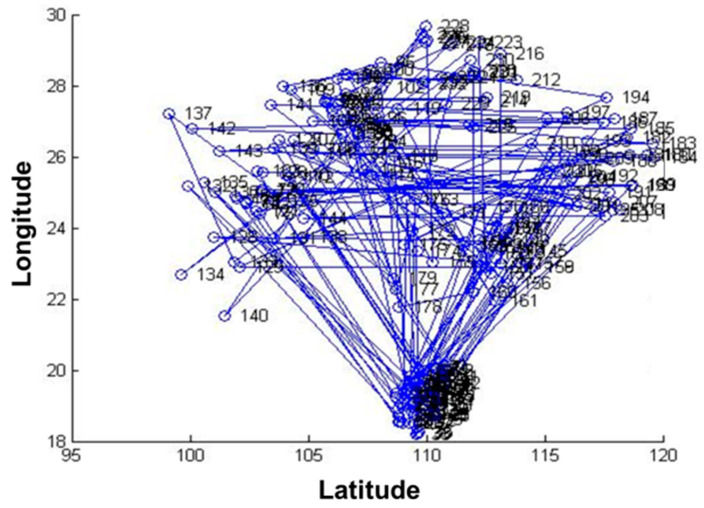
Running results of simulated annealing.

**Figure 9:**
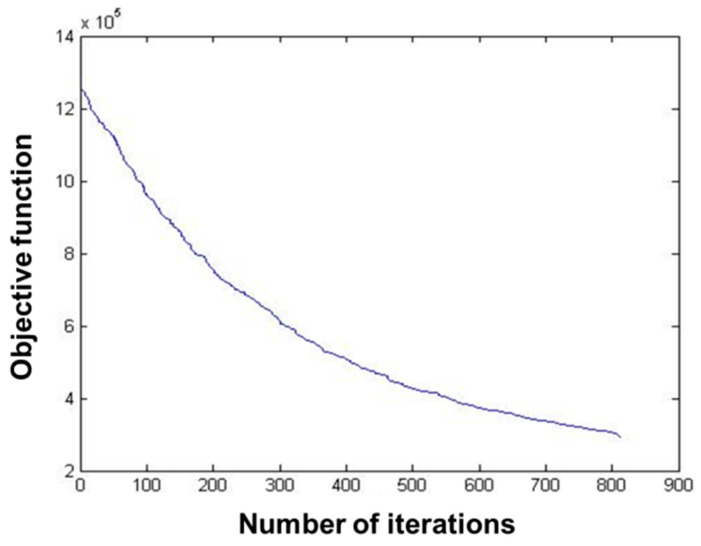
Convergence trajectory of simulated annealing.

The data of 235 pest monitoring points were imported into MATLAB software, and the SA was used for optimization analysis, and the results are shown in Figure 8 and Figure 9. The total number of iterations was set to 900, and the value of the objective function decreased slowly with the increase in the number of iterations, and its convergence trajectory was smoother than that of the GA, and it reached the minimum value at about 800 iterations.

The results showed that the transmission completion time of 235 pest monitoring points was 262.738048, 4.868012, and 17.842523 s, respectively, after optimizing the sensors’ data transmission path based on GA, PSO, and SA (Table 1). The transmission speed after optimization based on PSO was much higher than that of GA and SA. The objective function values of all three algorithms decrease with the increase in the number of iterations, the curves gradually converge, and the objective function values are close to the optimal. The convergence trajectory of the PSO has reached the optimal value at about five iterations, and the GA and SA reach the optimal value at about 900 and 800 iterations, respectively. The convergence speed of the PSO-based algorithm is higher than that of the GA and the SA, and the convergence trajectory of the GA has more transients. This show that the performance of the PSO algorithm is better than that of the GA and the SA in the optimization of sensors’ data transmission path for pest monitoring based on three algorithms.

## 4. Discussion

Internet technology is a new distributed sensors network and information transmission network integrating various sensing technologies, modern communication technologies, artificial intelligence, and automatic control. It has been widely used in insect monitoring and early warning, as well as agricultural product traceability, etc. [21,22]. Agricultural pests are characterized by a wide range of species, large numbers, wide distribution, and serious damage, which makes the monitoring and integrated prevention and control of agricultural pests particularly important. In the process of monitoring agricultural pests in the field, considering the mobility and random rows of agricultural pests, a large number of monitoring points need to be set up in the monitoring area, which facilitates the construction of a sensors network for pest monitoring and maximizes the collection of pest information [23]. However, the interconnection of multiple monitoring points means that the sensors’ data transmission from monitoring nodes to aggregation nodes may have a large amount of redundant data, making the monitoring network congested or even collapsing, resulting in failure to receive timely pest information. A total of 235 pest monitoring points were selected and the sensors’ data transmission paths were optimized based on three bionic intelligent algorithms (GA, PSO, and SA) in our study to improve the sensors’ data transmission efficiency for pest monitoring. The data transmission completion time was 262.738048 s after optimization using GA, 4.868012 s for PSO, and 17.842523 s for SA. The transmission time after optimization based on PSO was much lower than that of GA and SA, which shows that the PSO presents the best effect on the optimization of sensors’ data transmission path. Zhu et al. proposed a target recognition method based on PSO for data transmission path in wireless sensor networks, with an average delay of 1.8 μs. The transmission energy consumption and delay are reduced [24].

The GA is a computational model of the biological evolutionary process that simulates the natural selection and genetics mechanism of Darwinian biological evolution, the PSO is an intelligent algorithm designed by simulating the predatory behavior of a flock of birds, and the SA is derived from the principle of solid annealing. All three algorithms are heuristic algorithms, which are mathematical simulations of natural processes, and all are global optimization-seeking algorithms [25]. The GA has a strong global search capability and can jump out of the local optimum well, but it has a slow convergence, long running time, and insufficient local search capability; the PSO is simple to operate, and does not require complex behaviors such as population selection, crossover, and mutation, it runs fast but has the risk of falling into the local optimum solution; the SA has a strong local search capability, short running time, and poor global search capability [26,27]. Premalatha et al. proposed a hybrid algorithm PSO-GA based on GA, which applied the variation of GA to PSO and avoided the PSO to fall into the local optimal solution [28]. Da et al. proposed an improved PSO algorithm based on the SA technique, and the results showed that the SAPSO-based artificial neural network presents a better ability to escape from local optimum and is more effective than the traditional PSO-based artificial neural network [29]. A hybrid algorithm using a combination of GA and SA is proposed to solve the shortest path optimization problem of undirected networks, which prevents the occurrence of premature maturation, ensures the diversity of the population, and can effectively prevent the occurrence of falling into the local optimal search situation [30]. The three algorithms are pairwise combined into the hybrid algorithm, which can effectively complement the prematurity of GA, and the disadvantages of convergence speed decrease and similarity increase due to the random search algorithm. The sensors’ data transmission paths were optimized based on each of the three algorithms and compared in our study, and if the hybrid algorithm can be used in combination with the agricultural pest monitoring data transmission, the sensors’ data transmission path is further optimized.

In the process of agricultural pest monitoring, the performance of sensor nodes plays a decisive role in data transmission [31]. The speed of sensors’ data transmission for pest monitoring is guaranteed, and the data can be transmitted in real-time to develop pest control strategies in time after using PSO to optimize the path, but the sensor nodes are powered by batteries, which have limited energy and are extremely inconvenient to charge [32]. The lifetime of sensor nodes depends on the battery life, and excessive energy loss can cause the premature end of the agricultural pest monitoring network [33]. Therefore, while considering the transmission speed, the energy required for sensors’ data transmission also needs to be considered. Yang proposed a high-energy data transmission optimization algorithm-OTCEE based on tree-cluster topology, which reduces the energy loss of sensor nodes and delays the death of nodes [34]. Abhishek et al. developed an energy-saving scheme based on PSO optimization and GA optimization techniques, which solves the sensor node energy consumption fast [35]. Therefore, the efficiency of sensors’ data transmission for pest monitoring will be greatly improved if an algorithm can be designed to maximize the transmission speed and minimize the energy consumption.

## 5. Conclusions

In conclusion, this study uses three intelligent algorithms (GA, PSO, and SA) to optimize the data transmission paths of the sensors, and the optimization results are compared and analyzed. The results show that the optimized path based on PSO presents the shortest time used for transmitting data, and its minimum time is 4.868012 s. This study can provide a reference for improving the sensors’ data transmission efficiency for agricultural pest monitoring, and guarantee the development of real-time and effective pest control programs.

## Figures and Tables

**Table 1:** The elapsed time of three algorithms to complete the optimal path.

Types of Algorithms	Elapsed Time (s)
Genetic Algorithm	262.738048
Particle Swarm Optimization	4.868012
Simulated Annealing	17.842523

## Data Availability

The datasets generated during and/or analyzed during the current study are available from the corresponding author on reasonable request.
